# Long-Term Impacts of COVID-19 on Thyroid Health: Insights From Clinical Studies

**DOI:** 10.7759/cureus.71469

**Published:** 2024-10-14

**Authors:** Ria Syal, Jasmeet Kaur, Maheen Siddiqui, Hajera Amatul-Raheem, Cristhian Suarez, Nagavenkata Lova Surya Vamsi Avinash Bojanki, Sagar D Kapadia, Anil Kumar Yennam, Keerthi Kunchala, Sherien Metry, Umme Ruma

**Affiliations:** 1 Department of Biology, University of Waterloo, Cambridge, CAN; 2 Department of Medicine, Sapienza University of Rome, Rome, ITA; 3 Department of Cardiac Sciences, SINA-Health, Education and Welfare Trust, Karachi, PAK; 4 Department of Medicine, Deccan College of Medical Sciences, Telangana, IND; 5 Department of Medicine, Universidad San Francisco de Quito, Quito, ECU; 6 Department of Medicine, Siddhartha Medical College, Vijayawada, IND; 7 Department of Emergency Medicine, Jio World Center, Mumbai, IND; 8 Department of Medicine, Emilio Auguinaldo College, Manila, PHL; 9 Department of Internal Medicine, Sri Venkateswara Medical College, Tirupati, IND; 10 Department of Medicine, Medical University of Assiut, Asyut, EGY; 11 Department of Medicine, Rajeev Gandhi University of Health Sciences, Bengaluru, IND

**Keywords:** clinical trials, covid-19, endocrinology, long-term, management, thyroiditis

## Abstract

Background: COVID-19 emerged in December 2019 and rapidly became a global pandemic. It has since been associated with the progression of various endocrine disorders, including thyroid disease. The long-term effects of this interplay have yet to be explored. This review explores the relationship between COVID-19 and thyroid diseases, emphasizing thyroid gland function and the clinical implications for managing thyroid disorders in infected individuals.

Objectives: This narrative review intends to provide insight into the scope of research that future clinical studies may aim to address regarding the long-term effects of COVID-19 infection on thyroid health.

Methods: Keywords including “thyroid disease”, “COVID-19”, and “long-term” were used to search PubMed and Google Scholar for updated and relevant clinical research.

Results: COVID-19 affects the thyroid gland multifacetedly and includes direct viral invasion, immune-mediated damage, and hypothalamic-pituitary-thyroid axis disruption. Approximately 15% of COVID-19 patients experience thyroid dysfunction, which can present as thyrotoxicosis, hypothyroidism, or non-thyroidal illness syndrome (NTI). Noteworthy findings include inflammatory thyroiditis. Long-term effects, including those observed in children, include persistent hypothyroidism and exacerbated pre-existing thyroid-autoimmune conditions. Management of thyroid disorders in COVID-19 patients requires consideration: anti-thyroid drug (ATD) therapy used to treat hyperthyroidism in COVID-19 patients may need adjustment to prevent immunosuppression. Radioactive iodine (ROI) alternatives and interleukin-6 (IL-6) receptor antagonists could offer potential benefits and should be further explored.

Conclusion: Longitudinal follow-ups post-COVID-19 for patients with new and pre-existing thyroid disorders can improve disease outcomes. In addition, pathophysiological research on thyroid dysfunction in COVID-19 may help develop strategies to prevent and alleviate thyroid gland abnormalities post-COVID-19.

## Introduction and background

COVID-19, first identified in December 2019 as a viral pneumonia outbreak in Wuhan, China, soon became a global health emergency and pandemic due to its highly contagious nature, rapid spread, and novel characteristics. The causative agent, SARS-CoV-2, is an RNA virus related to the viruses responsible for SARS-CoV-1 (identified in 2003) and MERS (identified in 2012) [1]. While meticulous research has identified various endocrine disorders, including diabetes and obesity, influencing COVID-19 outcomes, emerging studies have indicated an inverse impact of COVID-19 on the endocrine system [2]. These studies suggest potential new-onset endocrinological dysfunctions involving the adrenal glands, thyroid, and testes [3,4]. Given the relatively recent emergence of this pandemic, it is crucial to study the long-term impacts across different organs and systems to anticipate potential chronic health conditions. Such insights can guide proactive measures to mitigate these impacts and improve long-term outcomes for COVID-19 patients. This article specifically focuses on COVID-19-induced thyroid gland abnormalities, emphasizing its potential influence on the outcomes of thyroid diseases in infected individuals through its association with thyroid dysfunction.

## Review

Pathophysiological mechanisms in thyroid gland involvement in COVID-19

Studies have shown that COVID-19 and its vaccination are associated with thyroid dysfunction. COVID viral particles or their protein, which form components of vaccines, produce spike proteins that cause molecular mimicry to the angiotensin-converting enzyme (ACE) receptors and trigger the renin-angiotensin-aldosterone (RAAS) system, which may be the fundamental cause of thyroid dysfunction and other endocrinology disorders [5]. Thyroid abnormalities have been described in about 15% of the patients affected with mild to moderate COVID-19 [6]. 

There are several theories explaining the influence of COVID-19 on the thyroid gland. The thyroid gland expresses many ACE2 and transmembrane protease serine 2 (TMPRSS2) receptors, which are used by SARS-CoV-1 and SARS-CoV-2 to enter and infect the host cells [7]. SARS-CoV-2 can also generate an immune response by activating Th1/Th17 lymphocytes causing the release of interleukins 1-6 and tumor necrosis factor α creating a cytokine storm [8]. Another plausible mechanism is the direct invasion and infection of the thyroid gland by coronavirus from the upper respiratory tract, evidenced by the extensive apoptosis observed on the gland's histological examination of the gland [9].

**Figure 1:**
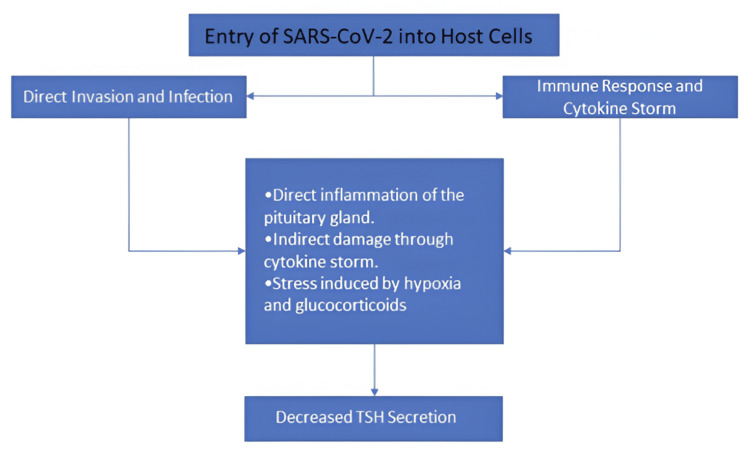
Effects of SARS-CoV-2 on pituitary function and the corresponding effect on its TSH secretion TSH: thyroid-stimulating hormone

**Figure 2:**
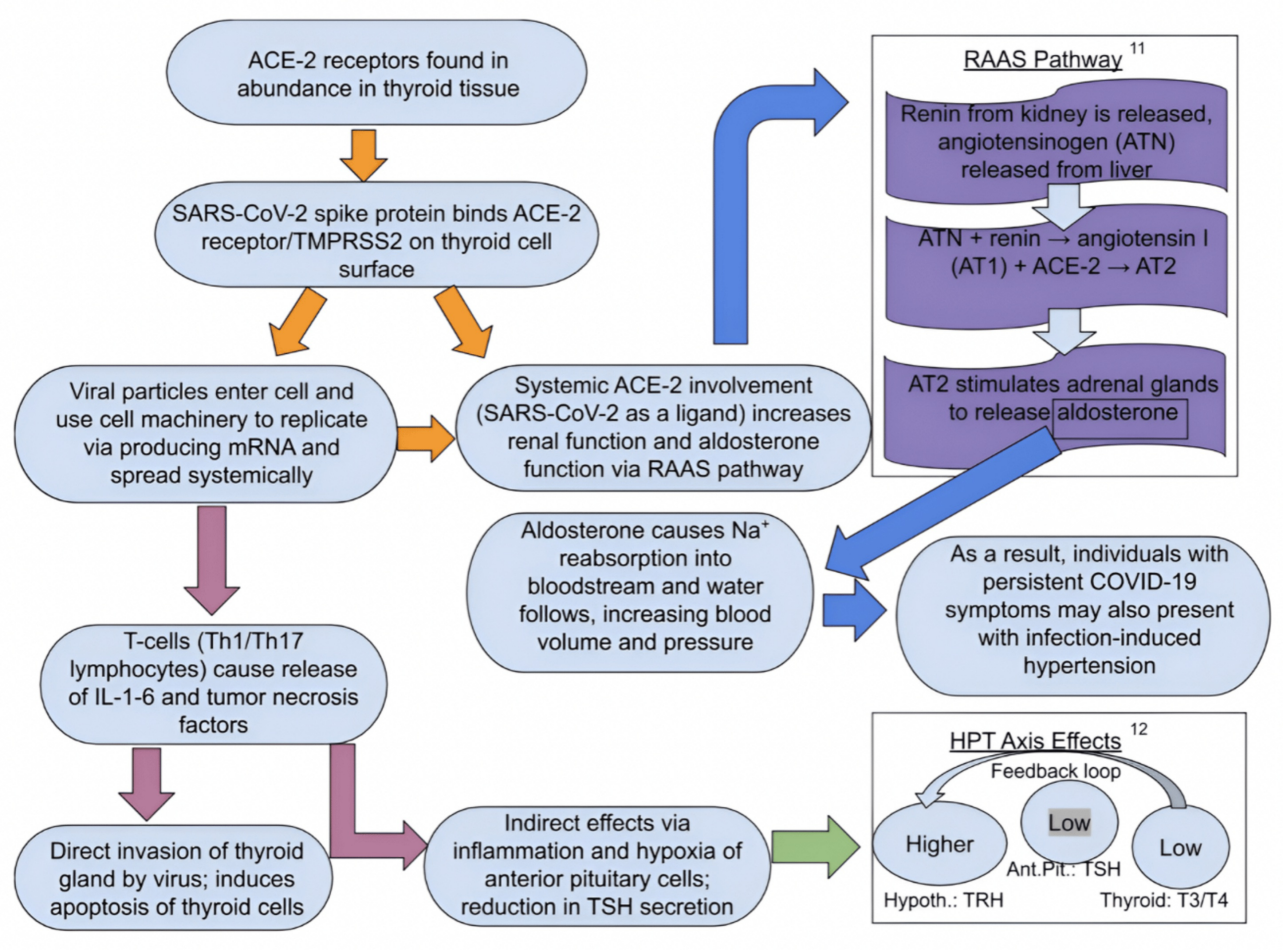
Pathophysiological methods of entry of SARS-CoV-2 and its associated systemic effects RAAS pathway and HPT axis effects [11,12] RAAS: renin-angiotensin-aldosterone system; ACE-2: angiotensin-converting enzyme-2; TMPRSS2: transmembrane protease serine 2; TSH: thyroid-stimulating hormone

In addition to its direct impact on the thyroid gland, coronavirus also causes a decrease in thyroid-stimulating hormone (TSH) secretion (Figure 1) by affecting TSH-secreting cells in the pituitary in different ways: direct inflammation of the pituitary gland, stress induced by hypoxia and glucocorticoids [10]. This results in reduced thyroid gland stimulation. Thus, SARS-CoV-2 affects the thyroid gland function directly and through the hypothalamic-pituitary-thyroid axis causing three main thyroid disorders: thyrotoxicosis, hypothyroidism, and non-thyroidal illness syndrome (NTI). Figure 2 shows these concepts in a more detailed pathophysiological perspective. 

Thyroiditis in patients with COVID-19

One of the less commonly discussed but significant impacts of COVID-19 is its effect on the thyroid gland leading to thyroiditis. Thyroiditis encompasses a group of disorders characterized by thyroid gland inflammation, which can lead to temporary or permanent thyroid dysfunction. As mentioned earlier, the virus infects thyroid cells via ACE2 receptors and produces an inflammatory response that may also explain thyroiditis. 

**Figure 3:**
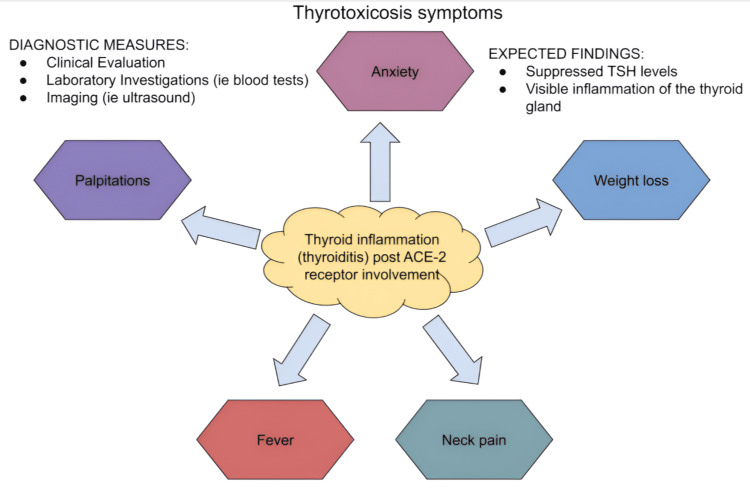
Thyrotoxicosis symptoms, diagnostic measures, and expected findings in patients presenting with thyrotoxicosis

Patients who develop thyroiditis after infection with COVID-19 often present with a variety of symptoms, including fever, neck pain, and symptoms of thyrotoxicosis, which include palpitations, anxiety, and weight loss. In some cases, thyroiditis can be silent, with no obvious symptoms, and is only detected through laboratory tests showing abnormal thyroid function [13]. Diagnosing thyroiditis in COVID-19 patients involves a focused clinical evaluation, laboratory investigations, and imaging. Laboratory tests typically show abnormal thyroid function, such as elevated TSH levels in hypothyroidism or, as shown in Figure 3, suppressed TSH levels in thyrotoxicosis. Imaging studies, such as ultrasound, can reveal inflammation and structural changes in the thyroid gland [14]. Management of thyroiditis in patients with COVID-19 depends on the severity of symptoms and the underlying thyroid function. Treatment options may include anti-inflammatory medications, beta-blockers for thyrotoxicosis symptoms, and thyroid hormone replacement for hypothyroidism. Close monitoring of thyroid function is essential to adjust treatment and identify any recurrence of symptoms [15].

Differences and similarities emerge when comparing thyroiditis in COVID-19-infected individuals with non-COVID-19 patients. COVID-19-related thyroiditis often presents with more severe systemic symptoms and a greater likelihood of recurring symptoms post-infection. These differences underscore the importance of tailored management strategies for these patients and highlight areas for future research, such as understanding the long-term impacts of COVID-19 on thyroid function and the development of targeted therapies [16].

Subacute thyroiditis (SAT) is an uncommon inflammatory thyroid disorder, often following a viral infection. Traditionally linked to viruses such as enterovirus, coxsackievirus, mumps, and measles, SAT has gained recent attention due to its association with the novel coronavirus [17]. Thyroiditis is characterized by an initial hyperthyroid phase, which may transition to hypothyroidism before normalizing.

We searched online databases, including PubMed/Medline and Google Scholar, for articles published until June 2024. We included case reports and case series about SAT in COVID-19 patients and tried to identify the long-term effects on thyroid function. A total of 40 articles including case reports and case series were found with 95 COVID-19 patients who developed SAT as a complication [15,17-50].

The findings of SAT in COVID-19 patients showed a resemblance with other cases of classic SAT. Our literature search revealed that most patients presenting with SAT were women. The main presenting symptom was anterior neck pain (69%), followed by fever (54%), general asthenia and malaise (38%), and palpitations (30%), the findings which are similarly reported by other reviews [19]. Most of the cases had no prior thyroid dysfunction or the patient history was not reported appropriately. The symptoms of SAT can present simultaneously with symptoms of COVID-19 disease or several days/weeks after. In the reviewed cases, treatment was successful, and outcomes were similar across patients. Elevated erythrocyte sedimentation rate (ESR) or C-reactive protein (CRP) was common, with ESR increasing in most cases. Low TSH levels along with high free T3 and T4 levels were observed in the initial phase of the illness. Thyroid ultrasonography and radionuclide scanning were used in most cases for diagnosis, showing classic sonographic findings of SAT and significantly decreased radionuclide uptake. Many SAT cases in COVID-19 patients reported elevated thyroid autoantibodies (anti-TPO, anti-Tg, and TRAb), which is uncommon in classic SAT [20]. Prednisone was the mainstay of the treatment for SAT in most of these patients along with nonsteroidal anti-inflammatory drugs (NSAIDs) and Beta-blockers if required [21]. 

Most patients recovered completely within a few days to weeks with their thyroid function tests and inflammatory markers returning to the baseline on follow-up with no long-term adverse effects [15,19,20,22-29,32-43,45-52]. However, some cases of hypothyroidism in initial follow-up months have also been reported, and treatment with levothyroxine started [18,21,26,28,53,54]. These findings are summarized in Table 1. Most cases reported in the literature do not report long-term follow-up after complete recovery from COVID-19-associated SAT. The temporal association between thyroid issues following COVID-19 infection and the presence of antithyroid antibodies suggests that COVID-19 may impact the immune system and thyroid gland. Therefore, long-term follow-up of patients with SAT is needed, as primary hypothyroidism can be an important long-term sequela in these cases.

**Table 1:** Long-term complications of SAT due to COVID-19 disease ESR: erythrocyte sedimentation rate; TPO: thyroid peroxidase; TRAb: TSH-receptor antibodies; SAT: subacute thyroiditis; NSAID: nonsteroidal anti-inflammatory drug; FT4: free thyroxine; FT3: free triiodothyronine

Authors	Age/sex	SAT symptoms	Thyroid lab tests	Thyroid ultrasound	Treatment		Long-term outcome
Chong, et al. [27]	49/M	Pain in the anterior neck and tenderness, fatigue, chills, palpitations, postural tremors, heat Intolerance, anorexia, and weight loss	FT4-2.3, FT3-202, TSH<0.01, and ESR-31	Diffusely hypoechoic thyroid	Oral aspirin and propranolol for a week		Hypothyroidism after three weeks that was treated with levothyroxine
Chakraborty, et al. [54]	51/M	Neck pain, high-grade fever, tachycardia, increased frequency of stools, and diffuse thyroid enlargement	FT4-20.11, FT3-2.88, TSH-<0.00, and ESR-110	Diffuse bilateral thyroid enlargement with hypoechogenicity, Increased vascularity, and a solitary nodule in each lobe	Propranolol 40 mg/day and oral Prednisolone 30 mg/day with Tapering over one-month		Hypothyroidism after a month that was treated with oral levothyroxine (50 μg/day)
Brancatella, et al. [15] (case series-15 cases)	Mean age- 34, F>M	Bilateral neck pain in 16 (89%) and fever in 17 (94%)	FT4-24.3 (4.6), FT3-5.8 (1.7), FT4/FT3-4.3 (1.2), TSH-0.1 (0.2), ESR -43 (45), CRP- 3.8 (2.2), and Tg-69 (70). Patients with positive TGabs (%)-62 (31) Patients with positive TPOabs (%) 27 (14)	Typical for SAT	Steroids and NSAID		Hypothyroidism in 13 of 15 patients
Brancatella, et al. [15]	29/F	Neck pain, fever, and palpitations	FT4-31, FT3-8.9, TSH-<0.001, Tg- 80 Tgab-pos Tpoab-neg Trab-neg ESR-110	Hypoechoic areas and reduced vascularization	Prednisone 25 mg/day with taper, propranolol 40 mg/day		Symptoms resolved completely within three weeks. Subclinical hypothyroidism occurred after 6 weeks
Brancatella, et al. [15]	29/F	Neck pain radiating to the jaw and right ear, palpitations, tachycardia, and sweating	NR	Diffuse enlarged gland, with multiple hypoechoic areas and absent vascularization at color doppler	Ibuprofen (600 mg/day)		Subclinical hypothyroidism
Feghali, et al. [26]	41/F	Palpitations and insomnia	FT4-1.9 ng/dL. TSH-<0.01, Tg-2.4, Tgab-3, Tpoab-69, and Trab-1		Initial management NR, levothyroxine 112 μg daily for the hypothyroid phase		Hypothyroidism after 3 weeks levothyroxine was started
Mondal, et al. (case series-11 cases) [21]	Mean age- 44, F=7, M=4	Palpitations, tremor, diarrhea, fever, congestive heart failure, and neck pain and tenderness	FT4-1.59-3.61, FT3-0.73-8.6, and TSH-0.02-000.5	Diffuse thyromegaly (n=7, 63.6%), hypoechoic areas (n=11, 100%), and/or reduced vascularity (n=4, 36.4%	Beta-blockers 11, NSAIDs=2, Glucocorticoids=4		Euthyroidism=9, subclinical hypothyroidism=1, overt hypothyroidism=1
Osorio, et al. [18]	64/M	Distal tremor with generalized diaphoresis and impalpable thyroid gland	FT4-1.85, FT3- 643.4, TSH-<0.01, Tgab-17, Tpoab-15, and Trab-0.56	Diffusely enlarged micro-nodular thyroid gland	Atenolol 50 mg twice daily and prednisone 50 mg daily with tapering in 2 weeks		Primary hypothyroidism
San Juan, et al. [53]	47/F	Neck pain radiating to submandibular region	FT4-1.68, FT3-8.9, TSH-<0.05, Tgab-neg, Tpoab-neg, and Trab-neg	Enlarged right lobe with ill-defined hypoechoic area	Celecoxib		Overt hypothyroidism after 8 weeks that Was managed with levothyroxine

Long-term impact of COVID-19 on hypothyroidism and hyperthyroidism

A study that analyzed the prevalence of thyroid dysfunction in 287 COVID-19-positive patients found thyrotoxicosis and hypothyroidism in 58 (overt in 31) and 15 (overt in 2) patients [55]. Central hypothyroidism affecting the hypothalamus or pituitary was found in 2%-6% of COVID-19 hospitalized patients by Chen et al., indicating acute and transient effects of COVID-19 on the hypothalamus-pituitary-thyroid axis [56]. In addition to these immediate and transient effects, Szczerbiński et al. compared patients who were infected with COVID-19 to an age- and sex-matched control group before the pandemic to explore the long-term consequences of SARS-CoV-2 infection on the endocrine system [57]. The results showed significantly decreased levels of free triiodothyronine (FT3) and free thyroxine (FT4) and increased levels of TSH and thyroid peroxidase (TPO) antibodies in patients with COVID-19 six months after the initial infection suggesting autoimmune hypothyroidism [57]. According to a study conducted in Iran on 390 COVID‐19 patients, 21 were found to have hypothyroidism [58]. Another study compared patients with mild symptoms to those with severe pneumonia as a result of COVID-19 and showed overt hypothyroidism in three and overt thyrotoxicosis in eight patients in the latter group [59].

Not only is thyroid dysfunction associated with the acute and recovery phases of COVID-19 but also as a consequence of COVID-19 vaccination [59]. Post-COVID-19, patients may experience persistent or new-onset thyroiditis symptoms, including hypothyroidism characterized by fatigue, weight gain, and depression. Some patients may also report ongoing neck pain and tenderness. The recurrence of these symptoms can be challenging for patients and healthcare providers, highlighting the need for long-term monitoring and management [60]. Post-COVID thyroid function tests can provide insights into variations from baseline thyroid function. For patients with pre-existing thyroid abnormalities, these tests can guide adjustments to their treatment regimens, accommodating any changes that arise following infection.

A Chinese study of 79 patients a month after infection found lower thyroid volumes in males with higher initial SARS-CoV-2 viral loads, and 11 patients had thyroiditis [61]. Another study six months post-infection found smaller thyroid volumes in survivors compared to controls [62]. Similarly, studies also suggest that COVID-19 can aggravate pre-existing thyroid conditions. Mateu-Salat M et al. reported two cases of Graves’ disease following COVID-19 onset, one of whom had a history of Graves’ disease in remission for over 30 years, while the other had no pre-existing thyroid condition [60]. Additionally, three separate studies have documented a total of five cases of Graves’ disease emerging one to two months after COVID-19 infection. Among these cases, three individuals had a history of Graves’ disease in remission for several years, indicating that SARS-CoV-2 may trigger latent or new-onset autoimmune thyroid disorder [63]. Further illustrating the impact on thyroid function, the first case of thyroid storm due to SARS-CoV-2 in a Graves’ disease patient has been documented SARS-CoV-2 [64]. Another study, conducted in Hong Kong on 191 hospitalized COVID-19 patients, showed low TSH in 13.1% and high TSH and TPO antibody titer in only one patient [6]. A case of a 69-year-old female with myxedematous coma in the setting of COVID-19 is another example of thyroid abnormalities in COVID-19 [65]. These studies suggest that COVID-19 significantly alters thyroid function and could potentially exacerbate pre-existing thyroid abnormalities, particularly Graves’ disease. The impact on pre-existing conditions and the possible overlap that new-onset thyroid abnormalities may have with existing thyroid disorders leading to exacerbated conditions warrants a need for long-term evaluation of patients infected with COVID-19 in terms of thyroid health. Research into the long-term course of pre-existing thyroid conditions post-COVID-19 recovery is essential to ensure improved management and care for patients with thyroid diseases.

SARS-CoV-2 can result in long-term thyroid dysfunction evidenced by a study that showed persistent hypothyroidism in 7% of COVID-19 survivors [29]. Acute SARS-CoV-2 infection can also result in post-acute sequelae of SARS-CoV-2 infection (PASC), commonly known as long-COVID, with a multisystemic involvement [66]. The expression of ACE2 receptors on endocrine glands is suggestive of various endocrine disorders including diabetes, autoimmune thyroiditis, adrenal insufficiency, and infertility in men to be associated with post-COVID-19 disease [67]. Hence the potential for a long-term alteration in thyroid function is evident and may have major health implications as with other endocrine functions.

Lastly, drugs that are used in the treatment of COVID-19 can also affect the thyroid, as will be discussed later in this article [57]. Glucocorticoids decrease serum TSH levels by causing inhibition of thyrotropin-releasing hormone secretion in the hypothalamus, and TSH release from the pituitary and heparin increases serum-free T4 by displacing total T4 from thyroid-binding globulin [68]. Table 2 shows a summary of six studies that analyzed the long-term effects of COVID-19 on thyroid health.

**Table 2:** Long-term impact of COVID-19 on hypothyroidism and hyperthyroidism according to six clinical studies FT4: free thyroxine; FT3: free triiodothyronine

Study	Findings
Naguib R. [9]	Thyrotoxicosis: 58 patients (overt in 31); hypothyroidism: 15 patients (overt in 2)
Chen, et al. [56]	Central hypothyroidism in 2%-6% of hospitalized COVID-19 patients
Szczerbiński, et al. [57]	Decreased FT3 and FT4 levels and increased TSH and TPO antibodies in COVID-19 patients six months post-infection
Daraei, et al. [58]	21 patients found to have hypothyroidism
Güven, et al. [59]	Overt hypothyroidism: 3 patients (severe pneumonia group) and overt thyrotoxicosis: 8 patients (severe pneumonia group)
Mateu-Salat, et al. [60]	Persistent or new-onset thyroiditis symptoms in post-COVID-19 patients

In conclusion, not only does COVID-19 affect immediate thyroid function but it also leads to long-term thyroid complications underscoring the necessity for further research in this area.

COVID-19-associated endocrinopathies in the pediatric population 

Among the few clinical trials studying thyroid function changes in children with a history of COVID-19 infection, even fewer studies have aimed to find changes in thyroid health, upon contraction of COVID-19, for patients with a pre-existing thyroid condition. 

**Figure 4:**
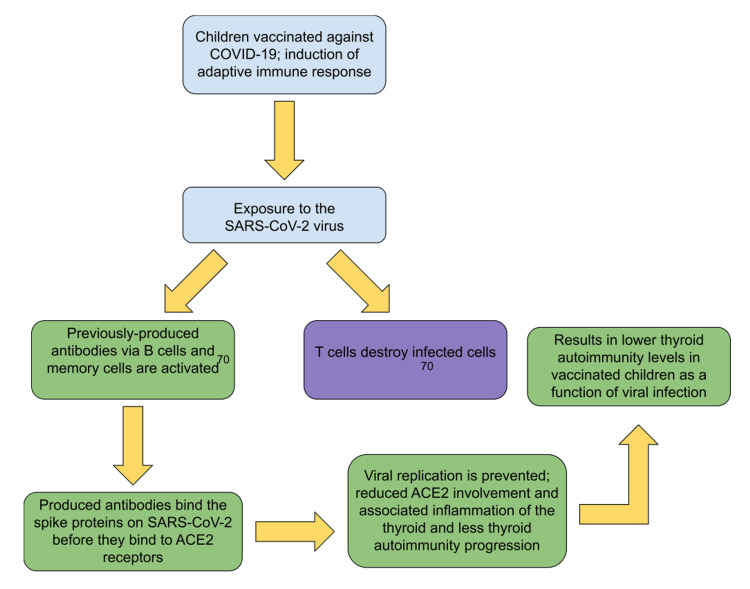
Immune response involved in developing viral particle recognition by pediatric host cells and the associated effects on thyroid autoimmunity B-cell and T-cell function [70]

In a study conducted by Herczeg et al. in 2023, researchers first confirmed previous COVID-19 history via polymerase chain reaction (PCR) and anti-spike protein serology [69]. Thyroid autoimmunity (TA) refers to autoimmune disorders affecting the thyroid gland and is an umbrella term for disorders including Hashimoto’s thyroiditis and Graves’ disease [28]. Children who had previously been vaccinated against COVID-19 showed a reduced rate of TA (5.7% TA post-pandemic) compared to those who had not been vaccinated before the contraction of the infection (6.8% TA post-pandemic). In the study, children were tracked for their thyroid function for 12.7 months and then re-assessed: two patients with TA showed a progression to general thyroiditis, confirmed via ultrasound, which suggests that in some individuals in the pediatric population, symptoms of thyroid dysfunction may take over a year to present following COVID-19 infection. Figure 4 shows the mechanism involved in providing COVID-19 immunity to children previously vaccinated against the virus.

As discussed earlier, the COVID-19 infection-causing virus, SARS-CoV-2, binds to ACE2 receptors on the thyroid gland, which implies that individuals with diminished thyroid function before contraction of the virus are at an increased risk of worsened thyroid abnormalities after the virus [71]. In a study conducted at a pediatric endocrine clinic in the United Kingdom, for children with thyroid abnormalities between January 1st, 2016 (pre-pandemic) and December 31st, 2021 (during/”post”-pandemic), the quantity receiving treatment for their disorder decreased post-pandemic, suggesting that some children may have had transient, but not chronic, thyroiditis associated with the COVID-19 virus, which later resolved by itself. 

While there have been no clinical studies indicating a positive correlation between pre-pandemic thyroid abnormalities in children and higher COVID-19 contraction rate or susceptibility to the virus, there is a potential link between pre-existing thyroid manifestations and a particular lowering of TSH when patients contract COVID-19, according to a clinical study done by Chen et al. in 2021. Greater decreases in values in thyroid function tests during the progression of COVID-19, including TSH and serum total triiodothyronine (TT3), were associated with higher COVID-19 severity [72]. While the trial did not take the pediatric population into explicit consideration, similar trials studying TSH and TT3 reductions in children with pre-existing thyroid pathologies are an open field for novel research regarding long-term COVID-19 influences. Summaries of three clinical studies that have aimed to analyze pediatric thyroid-related endocrinopathies as a function of COVID-19 are presented in Table 3.

**Table 3:** Pediatric endocrinopathies associated with COVID-19 according to three clinical studies TA: thyroid autoimmunity

Study	Findings	Conclusions
Herczeg, et al. [69]	Children vaccinated against COVID-19 showed lower rates of TA post-pandemic (5.7%) compared to unvaccinated children (6.8%). Two patients with TA progressed to thyroiditis confirmed by ultrasound over a 12.7-month follow-up period	Suggests potential delayed onset of thyroid dysfunction post-COVID-19 infection in some pediatric cases
McCowan, et al. [71]	Decrease in children receiving treatment for thyroid disorders post-pandemic, indicating transient thyroiditis that resolved	Suggests the transient nature of some COVID-19-related thyroid abnormalities in children
Chen, et al. [72]	Higher severity of COVID-19 is associated with greater decreases in thyroid function tests (TSH and TT3). Not specific to the pediatric population but highlights potential impacts on thyroid function in COVID-19 patients	Indicates a possible link between COVID-19 severity and thyroid hormone changes, calling for further research into pediatric populations with pre-existing thyroid conditions

Clinical implications and management considerations

Thus far, there has been no indication that poorly managed pre-existing thyroid diseases, including autoimmune pathologies such as Graves’ disease and Hashimoto’s thyroiditis, increase the probability of COVID-19 contraction. While there are several hypotheses regarding the few retrospective observational studies conducted by different research groups, there has not yet been an abundance of clinical trials entailing how contraction of COVID-19 can alter the management procedures of pre-existing thyroid conditions. 

Those with hyperthyroidism are typically prescribed anti-thyroid drug (ATD) therapy to reduce the levels of systemic thyroid hormone. ATDs have been shown to induce neutropenia as a rare side effect; neutropenia entails reduced counts of neutrophils, a type of white blood cell (WBC) crucial to fighting infections [73]. This suggests that when individuals with hyperthyroidism contract COVID-19, a viral infection, those being treated with ATD therapy may need to change their treatment regimen to manage their disease to prevent excessive difficulty fighting off the COVID-19 infection. A change in such management of hyperthyroidism for those with COVID-19 could include radioactive iodine (RAI) therapy; since COVID-19, it has been recommended by the Brazilian Committee for Nuclear Energy that the radiation intensity of this treatment be increased two-fold than what was previously administered, with no increase in the rate of hospitalization for side effects of the higher dosage [74].

According to an analysis performed by Lisco et al. in 2021, it was found that patients who suffered a more severe form of COVID-19 infection had reduced serum-FT3 hormone and FT3 is postulated to be needed to reduce the systemic inflammatory effects that interleukin-6 (IL-6) induces on cytokines. A leading cause of low FT3 levels is sepsis, which suggests that contracting COVID-19 may be correlated with potentially severe systemic inflammatory responses that also increase the overall intensity of the immune response to infections [75]. Thus, modifying/managing pre-existing thyroid diseases may entail T3-replacement therapy to prevent a more severe infection course from COVID-19 [75].

**Figure 5:**
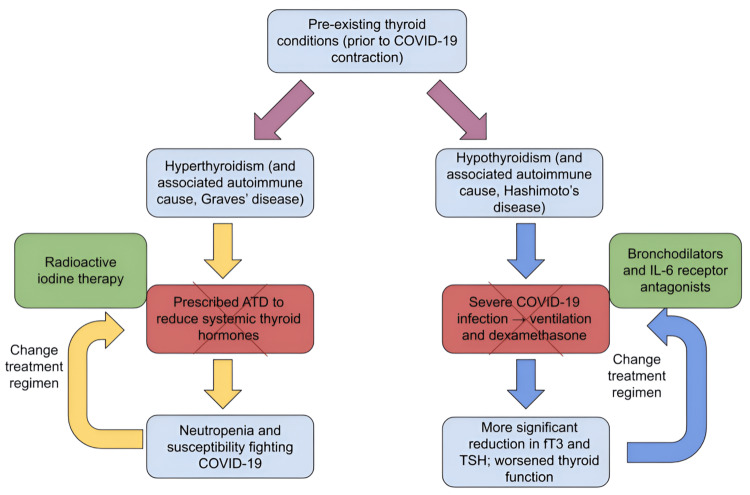
First-line treatments of thyroid disorders during COVID-19, related side effects, and potential changes to these pre-existing regimens The "X" through each red box represents a previously appropriate form of treatment for both hyperthyroidism and hypothyroidism that, when combined with COVID-19 infection, may produce adverse effects. Thus, the cross-outs represent incompatibility in the original treatment. The green boxes represent more suitable treatments for COVID-19 patients with a co-morbid thyroid disorder. IL-6: interleukin-6

It has been previously understood that corticosteroids, an example being dexamethasone, are beneficial for critically ill patients including those on ventilation, as frequently seen in very severe and end-stage phases of COVID-19 [75]. According to a randomized controlled double-blind crossover clinical trial conducted by Elston et al. in 2013, dexamethasone was noted to decrease both TSH and FT3 levels [76]. In this sense, treatments like dexamethasone may suppress hypothalamus-pituitary-thyroid function [76]. This suggests that for individuals with hypothyroidism who already have increased TSH levels to stimulate a moderately effective secretion of T3 and T4 (thyroid hormones), lowering TSH may potentially lead to an even more significant reduction in thyroid hormones. This may also suggest that for patients with thyroid disorders to manage their disease effectively throughout COVID-19, they should not be managing severe COVID-19 symptoms with corticosteroids. As previously discussed, medications like dexamethasone may be less invasive than mechanical ventilation in treating COVID-19 (beneficial as a first-line alternative due to overcrowding of medical centers and limited human resources); however, these treatments do not come without side effects for thyroid patients. Potential options for corticosteroids that may offer less harm to the thyroid gland include a combination of bronchodilators, monoclonal antibodies that recognize the COVID-19 spike protein and destroy such proteins, and IL-6 receptor antagonists such as sarilumab and tocilizumab [77]. As previously mentioned, IL-6 increases systemic inflammation; the use of IL-6 receptor antagonists as an alternative is supported by the fact that these may reduce immune reactions blown out of proportion upon contraction of COVID-19, thereby potentially reducing mortality rates. Figure 5 shows a visual representation of changes to treatment regimens for thyroid disorders that may be recommended upon contraction of COVID-19.

Observational studies have opened a window for more clinical trials and highlighted the need to investigate the evidence behind scientific claims. It is suggested that future research should study thyroid function tests when individuals change their COVID-19 treatment regimen, thyroid disorder treatment regimen, or both. This may provide insight into drug/procedure interactions, and if this data is collected from patients longitudinally, it could reveal the long-term risks or benefits of certain management techniques.

## Conclusions

In this narrative review, we have highlighted the physiological bases for the influence of the COVID-19 virus on thyroid function across different populations: the general population, pediatric patients, and individuals managing pre-existing thyroid conditions with specific medications. Recent clinical studies have explored the implications of COVID-19 on thyroid health, revealing a consensus in the scientific community that COVID-19 may transiently induce or exacerbate thyroid-related symptoms and thyroiditis. Additionally, alteration in thyroid gland activity could result in significant long-lasting alterations in thyroid functioning.

Moving forward, ongoing monitoring of thyroid hormones in patients with pre-existing thyroid diseases post-infection and recovery from COVID-19 could provide valuable insights into the long-term association between SARS-CoV-2 and thyroid gland function. The long-lasting nature of new-onset thyroid abnormalities must also be addressed as part of meticulous research in the future. Further research in this regard must be focused on long-term follow-up of patients post recovery from the infection delving deeper into the implications and pathophysiology of long COVID syndrome. Such understanding entails efforts directed toward a holistic approach to the longstanding aftermath of contracting the virus across all organ systems. The overlap between the pathophysiology of the coronavirus and the endocrinological consequences of the illness warrants a need for research evaluating possible interventions, post-COVID lifestyle modifications, and potential pre-emptive measures mitigating the long-term impacts that the infection may have on thyroid health. Vigilant detection and management of thyroid complications associated with COVID-19 are crucial for optimizing overall patient care and outcomes in the post-COVID-19 period. The findings discussed in this article emphasize the importance of implementing healthcare plans that concurrently consider viral infections and thyroid health to enhance patient outcomes.
